# The Right PPC Plays an Important Role in the Interaction of Temporal Attention and Expectation: Evidence from a tACS-EEG Study

**DOI:** 10.3390/biomedicines14020336

**Published:** 2026-01-31

**Authors:** Bingbing Fu, Kaishi Lin, Ying Chen, Junjun Zhang, Zhenlan Jin, Ling Li

**Affiliations:** MOE Key Lab for Neuroinformation, Brain-Computer Interface & Brain-Inspired Intelligence Key Laboratory of Sichuan Province, Center for Psychiatry and Psychology, School of Life Science and Technology, University of Electronic Science and Technology of China, Chengdu 610054, China; fubingbing@std.uestc.edu.cn (B.F.); kaishi.lin@std.uestc.edu.cn (K.L.); ying.chen@std.uestc.edu.cn (Y.C.); jjzhang@uestc.edu.cn (J.Z.); jinzl@uestc.edu.cn (Z.J.)

**Keywords:** temporal attention, temporal expectation, transcranial alternating current stimulation (tACS), event-related potential analysis, time-frequency analysis

## Abstract

**Background/Objectives:** Temporal attention and temporal expectation are two key mechanisms that facilitate perception by prioritizing information at specific moments and by leveraging temporal predictability, respectively. While their behavioral interaction is established, the underlying neural mechanisms remain poorly understood. Building on functional magnetic resonance imaging (fMRI) evidence linking temporal attention to parietal cortex activity and the role of alpha oscillations in temporal prediction, we investigated whether the right posterior parietal cortex (rPPC) may be involved in integrating these two processes. **Methods:** Experiment 1 used a behavioral paradigm to dissociate temporal expectation from attention across 600 ms and 1400 ms intervals. Experiment 2 retained only the 600 ms interval, combining behavioral assessments with electroencephalography (EEG), recording following transcranial alternating current stimulation (tACS) applied to the rPPC to probe neural mechanisms. **Results:** Experiment 1 showed an attention/expectation interaction exclusively at 600 ms: enhanced expectation improved response times under attended, not unattended, conditions. Experiment 2 replicated these behavioral and event-related potential (ERP) findings. Temporal attention modulated N1 amplitude: in attended conditions, the N1 was significantly more negative under high versus low expectation, while no difference was observed in unattended contexts. Anodal tACS over the rPPC reduced this N1 amplitude difference between high and low attentional expectation conditions to non-significance. Restricting analyses to attended conditions, paired-samples *t*-tests revealed that alpha-band power differed between high and low expectation under sham tACS, but this difference was absent under anodal tACS, which also attenuated the corresponding behavioral attention/expectation interaction effects. **Conclusions:** These findings provide suggestive evidence that the rPPC may be key to integrating temporal attention and expectation, occurring in early processing stages and specific to brief intervals.

## 1. Introduction

Temporal attention and expectation are two key cognitive mechanisms in time-related perceptual processing [1]. Temporal attention is an active cognitive mechanism that allocates processing resources to behaviorally relevant time points and enhances sensory input during these moments, analogous to spatial attention but in the temporal domain [1,2,3]. Temporal expectation refers to the brain’s ability to predict when events will occur by extracting temporal regularities from prior experience and environmental cues, representing a predictive capacity for temporal rhythms [4].

Independent manipulation of temporal attention and expectation is required to investigate their interaction mechanisms. Neural studies show that temporal expectation suppresses activity for predictable events, whereas temporal attention enhances responses to attended stimuli, revealing their opposing roles in perceptual processing [5,6,7]. However, most studies on temporal expectation and attention have either examined them in isolation or conflated their effects [8,9,10]. For example, temporal cueing paradigms often compare predicted and attended events with unexpected and unattended events, making it difficult to dissociate their respective contributions. A magnetoencephalography (MEG) study utilizing auditory temporal cueing demonstrated that independently manipulated temporal attention and expectation jointly modulate beta-band oscillations. Specifically, unattended expected tones reduced beta synchrony compared to unexpected tones—a phenomenon termed predictive suppression. Critically, this expectation-driven suppression effect was weakened when tones were attended, revealing a dynamic interaction between temporal attention and expectation in sensory processing [11]. These principles similarly apply to visual stimuli. By independently manipulating temporal attention and expectation through behaviorally validated metrics (session-wise precision and trial-wise hazard rates), the results demonstrated that higher temporal precision strengthened attentional benefits, yet surprisingly, such benefits diminished with delayed onset despite increasing stimulus probability. These findings reveal their cooperative interplay in optimizing visual performance [12]. The parietal and frontal cortices constitute key neural substrates for temporal orienting.

During temporal orienting tasks, robust activations are observed in the left parietal cortex, presupplementary motor area (preSMA), and frontal operculum (ventral premotor cortex). These regions exhibit functional specialization: the left parietal cortex governs motor responses at precise time points, the preSMA mediates interval estimation, while the frontal operculum participates broadly in temporal attention and timing behaviors [2]. In neglect patients, the degree of voxel-based damage in the right posterior parietal cortex (PPC) significantly correlates with impaired sustained spatial attention [13]. tDCS studies demonstrate that bilateral PPC differentially modulates temporal processing accuracy and variability, with cathodal stimulation of the right PPC specifically inducing time overestimation [14]. These findings collectively establish the pivotal role of the right parietal cortex in temporal processing.

Alpha oscillations serve as a neural signature for both temporal attention and temporal expectation. Temporal expectations generated by regular rhythmic events modulate ongoing alpha oscillations, concentrating cortical excitability dynamics within the anticipated target onset window. Moreover, alpha desynchronization intensity positively correlates with temporal precision of expectation [15]. When predictive cues about visual target timing are provided to participants, temporal expectations modulate the phase of ongoing alpha-band oscillations, shifting them toward an optimal phase state for visual processing. This constitutes a top-down control mechanism for visual processing guided by temporal predictions [16]. Alpha oscillations implement a tripartite gating system for temporal attention: amplitude suppression induces global thalamocortical inhibition, negative phase constrains local integration windows, and frontoparietal coupling allocates resources to internal processing—converging to impose perception failures during the 200–500 ms Attentional Blink window. This model reconciles neurophysiological (thalamocortical loops), computational, and behavioral dimensions of temporal attention [17].

In the present study, we aim to dissociate the contributions of temporal attention and expectation by employing a factorial experimental paradigm that independently manipulates both mechanisms across long (1400 ms) and short (600 ms) interval conditions. We will probe the causal role of the right posterior parietal cortex (PPC) in their interaction by perturbing neural activity with 10Hz alpha-band transcranial alternating current stimulation (alpha-tACS). Furthermore, we acquired EEG data to investigate the neural mechanisms underlying the interaction effects.

## 2. Materials and Methods

### 2.1. Experiment 1

#### 2.1.1. Participants

A total of 26 participants were initially recruited, with 23 retained for final analysis (14 females, 9 males; mean age = 22.09 years; all right-handed; all were undergraduate or graduate students from the University of Electronic Science and Technology of China). Three participants were excluded due to accuracy rates below 80%. All participants received monetary compensation upon completion, and the study was approved by the Ethics Committee of the University of Electronic Science and Technology of China.

#### 2.1.2. Stimuli and Task

**Figure 1:**
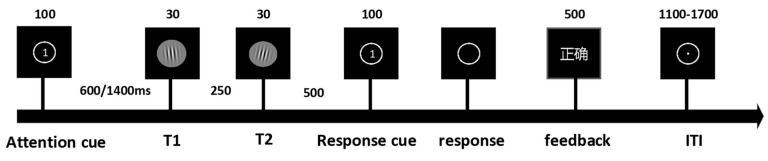
The experiment comprised 4 blocks (128 trials per block) presented in randomized order. Independent variables included temporal expectation: Certain (100%) vs. Uncertain (50%). Temporal attention: Valid cue vs. Neutral cue. Time intervals: Short (600 ms) vs. Long (1400 ms). Three types of attention cues were implemented: (1) Cue 1: Valid cue directing attention to the first target (32 trials); (2) Cue 2: Valid cue directing attention to the second target (32 trials); (3) Cue 0: Neutral/non-informative cue (64 trials). The attention cue screen presented numerical prompts to guide participants’ attention toward T1 or T2. The response cue indicated that participants could perform key-press responses after the main stimulus display disappeared. The feedback screen was presented in Chinese, displaying corresponding textual prompts to indicate whether participants’ behavioral responses were correct or incorrect.

We designed experimental paradigms informed by the established literature on the temporal attention/expectation interaction [3,12]. Target stimuli consisted of ten Gabor patches characterized by Gaussian envelopes (σ = 0.3° visual angle), spatial frequency of 4 cycles per degree, and 100% Michelson contrast, with orientations offset clockwise or counterclockwise from the vertical axis (5°, 15°, 20°, 25°, 30°) and randomized spatial phases. Each trial initiated with a 500 ms central fixation period followed by a 100 ms attentional cue. After a 600 ms or 1400 ms delay interval, two target stimuli were presented consecutively, followed by a 100 ms response prompt. Upon prompt disappearance, participants responded as rapidly as possible using index fingers on ‘F’ (counterclockwise) or ‘J’ (clockwise) keys. A 500 ms accuracy feedback was provided post-response, preceding a randomized 1100–1700 ms inter-trial fixation. The experiment comprised four blocks presented in a counterbalanced order across participants (Figure 1). Each block contained 64 valid-cue trials and 64 neutral-cue trials, with two blocks employing fixed delays (600 ms or 1400 ms) and two blocks incorporating randomized delays (600/1400 ms equiprobable). All procedures were implemented via E-Prime 3.0 (Psychology Software Tools, Pittsburgh, PA, USA).

#### 2.1.3. Behavioral Data Analysis

Data analysis excluded trials with incorrect responses and reaction times beyond three standard deviations from the mean. Mean accuracy rates and reaction times were computed per participant for each experimental condition. Statistical analyses were conducted using JASP (v0.19.3) [18], performing two-way repeated-measures ANOVAs with factors of attentional cue (valid/invalid) and temporal expectation (certain/uncertain) for both 600 ms and 1400 ms delay conditions. Significant main effects (*p* < 0.05) were followed by paired-samples *t*-tests. For pairwise comparisons lacking interaction effects, Bonferroni [19] and Holm [20] corrections were applied to control family-wise error rates.

### 2.2. Experiment 2

#### 2.2.1. Participants

We recruited an independent cohort of 32 participants (16 female, 16 male; mean age = 21.72 years; all right-handed) to control for practice and expectation effects. All participants were undergraduate or graduate students from the University of Electronic Science and Technology of China, received monetary compensation, and the study was approved by the university’s Ethics Committee.

#### 2.2.2. Stimuli and Task

The apparatus, stimuli, and procedure remained identical to the first experiment, with the exception that the 1400 ms high-expectation condition was omitted. The experiment retained three blocks, each comprising 128 trials. A within-subject crossover design was implemented wherein all participants underwent both anodal and sham tACS conditions. The stimulation sequence was counterbalanced across participants using randomized block allocation. To mitigate carry-over effects, sessions were separated by a minimum 72 h washout period.

#### 2.2.3. tACS Set Up

Based on previous findings linking the right parietal site (P4) to temporal orienting [21] and supported by evidence that 10 Hz tACS can selectively modulate endogenous rather than exogenous attention, we hypothesized that applying 10 Hz tACS over the temporally relevant right parietal cortex (P4) would effectively modulate temporal attention—which also relies on endogenous control mechanisms (voluntarily directing attention to specific points in time based on expectation) [22]. This approach provides a direct way to test the causal role of alpha oscillations in this region in temporal attention.

We chose anodal stimulation based on methodological continuity and current understanding of tACS mechanisms. Following a previous study that successfully modulated memory function using anodal HD-tACS over the left parietal cortex [23] and supported by the dominant hypothesis that anodal stimulation may depolarize cortical neurons—thereby enhancing excitability and neural synchronization—we selected an anodal protocol to increase (rather than suppress) neural activity in the right posterior parietal cortex.

For stimulation parameters, we adopted a well-established HD-tACS montage targeting the right posterior parietal cortex. The active electrode was placed at P4, with return electrodes covering P2, P6, CP4, and PO4 (International 10-20 system). Stimulation was delivered at 10 Hz (alpha frequency) with 1.5 mA intensity for 20 min, while electrode impedance was kept below 5 kΩ. In the sham condition, the current was ramped up to 1.5 mA at the start, immediately dropped to 0 mA, and then briefly raised again to 1.5 mA before being turned off at the end of the 20 min session [21,23,24,25,26,27].

#### 2.2.4. EEG Data Acquisition and Analysis

EEG data were recorded using a 64-channel WaveGuard™ 10-5 system (ANT Neuro, Enschede, The Netherlands). Signals were acquired at a sampling rate of 1000 Hz, using CPz as the reference electrode and GND as the ground. Horizontal electrooculograms (EOGs) were obtained from one electrode located at the outer canthus of the left eye. All electrode impedances were maintained below 10kΩ.

The EEG data were first filtered (0.1–30 Hz bandpass) and segmented into epochs time-locked to the cue screen onset (−200 to 800 ms). Incorrect trials were excluded from further analysis. The data were then referenced to the average of all channels and baseline-corrected using the 200 ms pre-stimulus interval. Channels with amplitudes exceeding ±100 μV were marked as bad and interpolated using spherical spline interpolation (on average, 2.2 channels [3%] under anodal and 2.1 channels [3%] under sham stimulation were interpolated per subject). Additionally, trials containing EEG activity exceeding ±100 μV were excluded. For all participants, more than 120 valid trials were retained per condition. Finally, Independent Component Analysis (ICA) was implemented via the EEGLAB toolbox to remove artifacts such as blinks and lateral eye movements (on average, 6.6 [10%] components under anodal and 7.1 [11%] components under sham stimulation were removed per subject). Based on a detailed visual inspection of the grand-average event-related potential (ERP) waveforms under both anodal and sham stimulation conditions across various experimental contexts formed by different levels of temporal attention and temporal expectation, we observed that the N1 component consistently emerged within the 140–200 ms post-stimulus interval over the parieto-occipital region. Based on this observation, this time window was identified as optimal for N1 analysis. Neuromodulation of the rPPC enhances interhemispheric functional connectivity, enabling synchronized regulation of contralateral brain activity [28]. Accordingly, the mean amplitude of the N1 component was calculated by averaging the voltage values within the 140–200 ms time window across a cluster of electrodes over the left parieto-occipital region, including PO5, PO7, O1, P1, P3, P5, and P7.

For time–frequency analysis, during the preprocessing stage, the data were filtered with a 0.1–48 Hz bandpass. Baseline correction was applied using the −500 to 0 ms interval, and epochs were segmented into −1500 to 3000 ms windows. All other steps remained consistent with the ERP preprocessing pipeline. Time–frequency transformation was performed using the FieldTrip toolbox (version 250523). A wavelet transform was applied to compute time–frequency representations under specific experimental conditions (frequency range: 0.5–30 Hz in steps of 0.25 Hz). Decibel (dB) conversion was then carried out using the pre-stimulus interval from −500 ms to −200 ms as the baseline.

#### 2.2.5. Behavioral Data Analysis

The same data exclusion criteria as in Experiment 1 were applied, whereby trials with incorrect responses and those with reaction times exceeding ± 3 standard deviations from the mean were removed. For both the anodal and sham conditions, separate two-way repeated-measures ANOVAs were conducted with attentional cue (valid, invalid) and temporal expectation (certain, uncertain) as within-subject factors. Statistical significance was defined as *p* < 0.05. Significant interactions were further examined via post hoc tests employing a Bonferroni correction.

## 3. Results

### 3.1. Experiment 1

**Figure 2:**
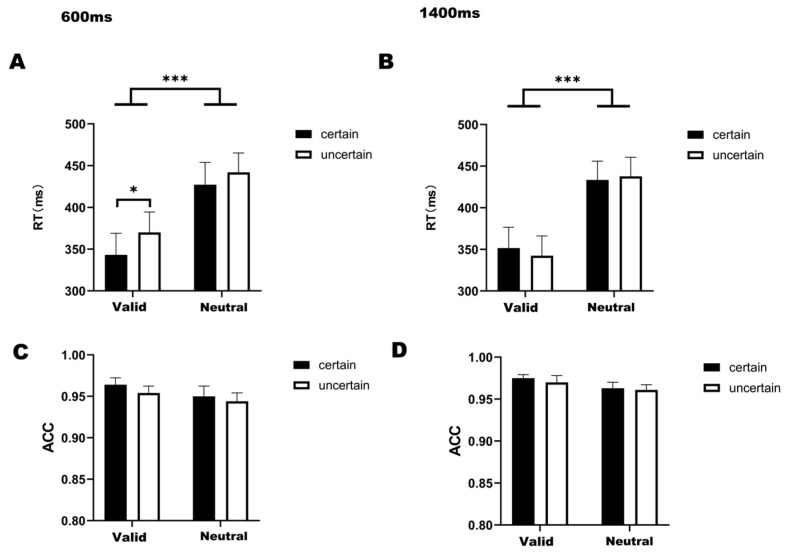
(**A**) Bar graph of RT under four conditions at 600 ms delay. (**B**) Bar graph of RT under four conditions at 1400 ms delay. (**C**) Bar graph of ACC under four conditions at 600 ms delay. (**D**) Bar graph of ACC under four conditions at 1400 ms delay. Note: * *p* < 0.05, *** *p* < 0.001, indicating significant differences between conditions.

For reaction times under the 1400 ms delay condition (Figure 2B), a significant main effect of temporal attention was observed (*F*_(1,22)_ = 55.167, *p* < 0.001, *η*^2^ = 0.544). The analysis revealed no statistically significant interaction between temporal attention and temporal expectation (*F*_(1,22)_ = 1.761, *p* = 0.198, *η*^2^ = 0.003). Bonferroni-corrected post hoc comparisons demonstrated significantly faster RTs in valid-certain trials compared to both neutral-certain (*t*(22) = −7.418, *p* < 0.001) and neutral-uncertain trials (*t*(22) = −5.314, *p* < 0.001). Similarly, valid-uncertain trials showed accelerated responses relative to neutral-certain (*t*(22) = −5.451, *p* < 0.001) and neutral-uncertain conditions (*t*(22) = −6.498, *p* < 0.001).

For the 600-ms condition (Figure 2A), a significant main effect of temporal attention was observed (*F*_(1,22)_ = 65.272, *p* < 0.001, *η*^2^ = 0.495), with no significant interaction between temporal attention and expectation (*F*_(1,22)_ = 0.955, *p* = 0.339, *η*^2^ = 0.003). Post hoc comparisons revealed faster reaction times in valid-certain trials compared to both neutral-certain (*t*(22) = −8.202, *p* < 0.001) and neutral-uncertain trials (*t*(22) = −6.818, *p* < 0.001). Similarly, valid-uncertain trials demonstrated accelerated responses relative to neutral-certain (*t*(22) = −3.727, *p* < 0.001) and neutral-uncertain conditions (*t*(22) = −5.751, *p* < 0.001). Holm-corrected comparisons further identified significantly faster responses in valid-certain versus valid-uncertain trials (*t*(22) = −2.492, *p* = 0.041).

For the ACC (Figure 2C,D), a two-way 2 (attention cue: valid/neutral) × 2 (temporal expectation: certain/uncertain) ANOVA conducted specifically under the 1400 ms delay condition (Figure 2D) revealed a significant main effect of temporal attention (*F*_(1,22)_ = 10.539, *p* = 0.004, *η*^2^ = 0.048). Subsequent post hoc comparisons showed no significant pairwise differences between any experimental conditions, and no significant interaction between temporal attention and temporal expectation was found in either the 600 ms (*F*_(1,22)_ = 0.291, *p* = 0.291, *η*^2^ = 0.002) or 1400 ms condition (*F*_(1,22)_ = 0.115, *p* = 0.738, *η*^2^ = 0.001).

### 3.2. Experiment 2

#### 3.2.1. Behavioral Result

*P*A comparison of participants’ self-reported pain ratings (0–10 points) under the two conditions was conducted. The results of a paired-samples *t*-test indicated no significant difference in pain ratings between the anodal tACS condition (Mean = 4.344, SD = 1.860) and the sham stimulation condition (Mean = 3.719, SD = 2.275), *t*(31) = −1.848, *p* = 0.074. Effect size evaluation revealed that this difference fell within the small effect range, Cohen’s d = −0.297. The 10 Hz tACS protocol employed in this experiment yielded no significant difference in measured subjective pain ratings compared to the sham stimulation condition. Notably, since a condition-guessing questionnaire was not administered to participants, we could only rely on the discrepancy in subjective pain perceptions to assess whether they could distinguish between anodal tACS and sham stimulation.

**Figure 3:**
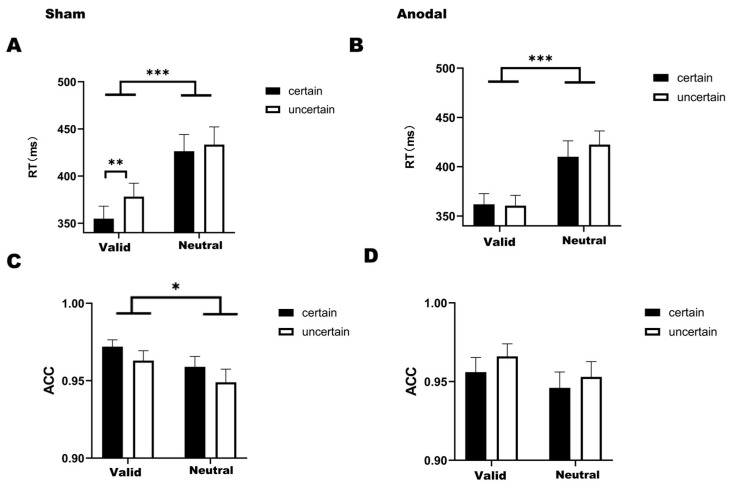
(**A**) Bar graph of reaction times under four conditions in the sham stimulation (Mean = 398.212, SD = 95.704). (**B**) Bar graph of reaction times under four conditions in the anodal stimulation (Mean = 388.743, SD = 77.607). (**C**) Bar graph of accuracy under four conditions in the sham stimulation (Mean = 0.961, SD = 0.038). (**D**) Bar graph of accuracy under four conditions in the anodal stimulation (Mean = 0.955, SD = 0.052). Note: * *p* < 0.05, ** *p* < 0.01, *** *p* < 0.001, indicating significant differences between conditions.

For reaction times under the sham condition (Figure 3A), the analysis revealed no statistically significant interaction between temporal attention and temporal expectation (*F*_(1,31)_ = 2.474, *p* = 0.126, *η*^2^ = 0.007). Significant main effects were observed for temporal attention (*F*_(1,31)_ = 46.635, *p* < 0.001, *η*^2^ = 0.448) and temporal expectation (*F*_(1,31)_ = 6.161, *p* = 0.019, *η*^2^ = 0.025). Post hoc comparisons showed that reaction times in the valid-certain were significantly faster than those in both the neutral-certain (*t*(31) = −6.269, *p* < 0.001) and the neutral-uncertain (*t*(31) = −6.186, *p* < 0.001). Similarly, reaction times in the valid-uncertain were faster than those in both the neutral-certain (*t*(31) = −5.219, *p* < 0.001) and the neutral-uncertain (*t*(31) = −5.627, *p* < 0.001). Furthermore, and consistent with the findings of the first experiment, reaction times in the valid-certain were significantly faster than those in the valid-uncertain (*t*(31) = −3.447, *p* = 0.010).

Under the anodal stimulation condition for reaction times (Figure 3B), temporal attention and temporal expectation did not interact significantly (*F*_(1,31)_ = 2.575, *p* =.119, *η*^2^ = 0.005). Only a significant main effect of temporal attention was found (*F*_(1,31)_ = 37.479, *p* < 0.001, *η*^2^ = 0.345). Bonferroni-corrected post hoc comparisons revealed that reaction times in the valid-certain were significantly faster than those in both the neutral-certain (*t*(31) = −4.648, *p* < 0.001) and the neutral-uncertain (*t*(31) = −5.805, *p* < 0.001) and that reaction times in the valid-uncertain were significantly faster than those in the neutral-uncertain (*t*(31) = −6.527, *p* < 0.001).

For accuracy, under the sham stimulation condition (Figure 3C), a two-way ANOVA revealed significant main effects of both temporal attention (*F*_(1,31)_ = 9.452, *p* = 0.004, *η*^2^ = 0.098) and temporal expectation (*F*_(1,31)_ = 5.052, *p* = 0.032, *η*^2^ = 0.049). Bonferroni-corrected post hoc comparisons indicated that accuracy in the valid-certain was significantly higher than in both the neutral-certain (*t*(31) = 2.957, *p* = 0.035) and the neutral-uncertain (*t*(31) = 3.515, *p* = 0.008). We found no significant interaction between temporal attention and temporal expectation (*F*_(1,31)_ = 0.018, *p* = 0.895, *η*^2^ < 0.001).

Under the anodal stimulation condition (Figure 3D), significant main effects were found for temporal attention (*F*_(1,31)_ = 5.696, *p* = 0.023, *η*^2^ = 0.064) and temporal expectation (*F*_(1,31)_ = 4.150, *p* = 0.050, *η*^2^ = 0.035). Bonferroni-corrected post hoc comparisons demonstrated that accuracy in the valid-uncertain was significantly higher than in the neutral-certain (*t*(31) = 2.902, *p* = 0.041). We found no significant interaction between temporal attention and temporal expectation (*F*_(1,31)_ = 0.119, *p* = 0.732, *η*^2^ = 0.001).

#### 3.2.2. ERP Result

**Figure 4:**
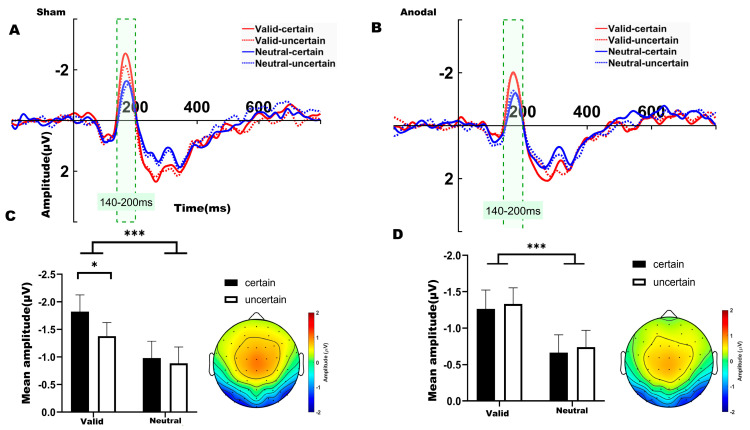
(**A**) ERP waveforms in the left parieto-occipital region (electrodes: P7, P3, P5, PO5, PO7, O1) under sham stimulation. (**B**) ERP waveforms in the left parieto-occipital region under anodal stimulation. (**C**) Average N1 waveforms (time window: 140–200 ms) under sham stimulation. (**D**) Average N1 waveforms (time window: 140–200 ms) under anodal stimulation. Note: * *p* < 0.05, *** *p* < 0.001, indicating significant differences between conditions.

For the N1 component, a two-way ANOVA was conducted to examine the effects of temporal expectation and temporal attention under each stimulation condition (sham, anodal), comparing the four experimental conditions. Over the left parieto-occipital region under sham stimulation (Figure 4A), significant main effects were observed for both temporal attention (*F*_(1,31)_ = 21.910, *p* < 0.001, *η*^2^ = 0.202) and temporal expectation (*F*_(1,31)_ = 5.658, *p* = 0.024, *η*^2^ = 0.033). We found no significant interaction between temporal attention and temporal expectation (*F*_(1,31)_ = 1.537, *p* = 0.224, *η*^2^ = 0.014). Bonferroni-corrected post hoc comparisons revealed that the mean amplitude in the valid-certain condition was significantly more negative than that in both the neutral-certain condition (*t*(31) = −4.041, *p* = 0.002) and the neutral-uncertain condition (*t*(31) = −5.270, *p* < 0.001). Consistent with the behavioral results, a significant difference was also found between the valid-certain and valid-uncertain conditions (*t*(31) = −2.895, *p* = 0.041), with the valid-certain condition exhibiting a larger (more negative) N1 amplitude. The mean N1 amplitudes under sham stimulation are summarized in Figure 4C.

Under anodal stimulation (Figure 4B), only a significant main effect of temporal attention was identified for the N1 amplitude (*F*_(1,31)_ = 15.803, *p* < 0.001, *η*^2^ = 0.156). We found no significant interaction between temporal attention and temporal expectation (*F*_(1,31)_ = 4.59 × 10^−4^, *p* = 0.983, *η*^2^ < 0.001). Bonferroni-corrected post hoc comparisons indicated that the valid-certain condition differed significantly from the neutral-certain condition (*t*(31) = −2.966, *p* = 0.035). Additionally, the valid-uncertain condition differed significantly from both the neutral-certain (*t*(31) = −3.196, *p* = 0.019) and neutral-uncertain conditions (*t*(31) = −4.248, *p* = 0.017). The mean N1 amplitudes under anodal stimulation are summarized in Figure 4D.

#### 3.2.3. Time–Frequency Results

**Figure 5:**
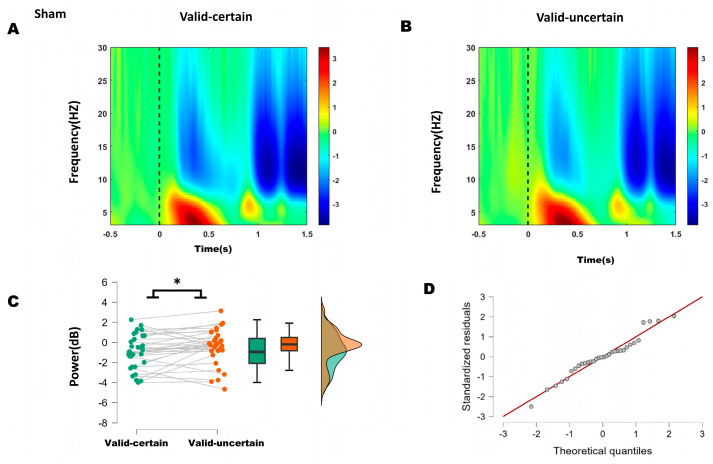
(**A**) Time–frequency representations in the parieto-occipital region (electrodes: PO5, PO7, O1, P1, P3, P5, P7, PO6, PO8, O2, P2, P4, P6, P8) under sham stimulation. (**B**) Time–frequency representations under anodal stimulation. (**C**) Comparison of mean alpha-band energy (200–250 ms time window) between valid-certain (Mean = −0.911, SD = 1.687) and valid-uncertain (Mean = −0.424, SD = 1.753) conditions using paired-sample *t*-tests. Green boxes and points represent the valid-certain condition; orange boxes and points represent the valid-uncertain condition. The panel progresses from showing individual data and means (left), to robust summary statistics (middle), and finally to detailed distribution shapes (right), collectively providing a comprehensive view of the significant difference between the two conditions. (**D**) Q-Q plot assessing normality of the difference distribution. Note: * *p* < 0.05, indicating significant differences between conditions.

**Figure 6:**
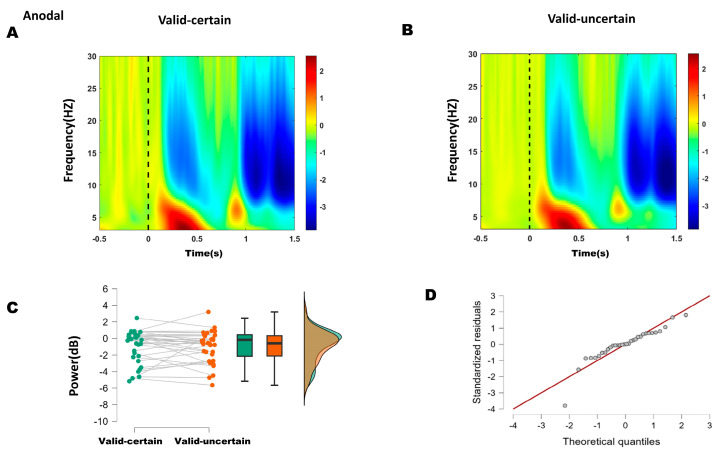
(**A**) Time–frequency representations in the parieto-occipital region (electrodes: PO5, PO7, O1, P1, P3, P5, P7, PO6, PO8, O2, P2, P4, P6, P8) under anodal stimulation. (**B**) Time–frequency representations under anodal stimulation. (**C**) Comparison of mean alpha-band energy (200–250 ms time window) between valid-certain (Mean = −0.941, SD = 1.963) and valid-uncertain (Mean = −1.007, SD = 1.931) conditions using paired-sample *t*-tests. Green boxes and points represent the valid-certain condition; orange boxes and points represent the valid-uncertain condition. The panel progresses from showing individual data and means (left), to robust summary statistics (middle), and finally to detailed distribution shapes (right). (**D**) Q-Q plot assessing normality of the difference distribution.

Both behavioral and ERP results indicated that the differences occurred specifically between the valid-certain and valid-uncertain conditions. The time-frequency representations for sham and anodal stimulation are shown in Figure 5A,B and Figure 6A,B, respectively. Accordingly, we performed paired-sample *t*-tests to compare the mean alpha-band power in the 200–250 ms time window over bilateral parieto-occipital regions under valid-certain versus valid-uncertain conditions, separately for sham and anodal stimulation. The differences between conditions were first assessed for normality.

For the sham condition, the difference between valid-certain and valid-uncertain wabas normally distributed (Shapiro–Wilk, *p* = 0.696); see Q-Q plot in Figure 5D. A Student’s *t*-test was therefore applied and, as Figure 5C, revealing a significant difference between conditions: alpha power under high attentional expectation was significantly lower than under low attentional expectation (−0.911 < −0.424, *t*(31) = −2.164, *p* = 0.038).

For the anodal condition, the difference between high and low attentional expectation was not normally distributed (Shapiro–Wilk, *p* = 0.001; see Q-Q plot in Figure 6D). The Wilcoxon signed-rank test indicated no significant difference in alpha power between high and low attentional expectation (*W* = 218, *p* = 0.400) (Figure 6C).

These results suggest that anodal stimulation reduced the difference in alpha energy between high and low attentional expectation conditions.

## 4. Discussion

Behavioral results from the first experiment demonstrated a significant main effect of temporal attention regardless of the delay interval duration. Building upon the temporal cueing paradigm, this study further dissociated temporal expectation from temporal attention. Under the long-delay condition, a significant difference was observed between valid and neutral attentional cues, with valid cues facilitating performance. This indicates that, similar to spatial attention, temporal attention also produces a significant cueing effect, aiding in the rapid allocation of attention to task-relevant temporal points [27,29,30,31,32,33,34].

The interaction between temporal attention and temporal expectation was only observed under short-delay conditions. This finding aligns with previous studies using temporal cueing paradigms that did not dissociate attention from expectation: a facilitatory effect of temporal cues was found under short delays, whereas no reaction time difference between valid and invalid cues was shown under long delays [5,27,35,36,37]. The brain’s efficient reallocation of attention makes it difficult to detect temporal attention effects over longer SOAs [2,4,5]. This adaptive reorienting ability also accounts for the absence of interaction effects between temporal attention and expectation over long intervals.

In the second experiment, sham stimulation replicated the behavioral results of the first experiment. Even after removing the 1400 ms condition, interactions between temporal attention and expectation were observed: under attended conditions, reaction times between high and low expectation differed significantly, whereas no such difference was found under unattended conditions. A similar pattern was observed in the N1 component—the mean amplitude under high attentional expectation was significantly more negative than under low expectation. However, these interactions were attenuated under anodal stimulation.

Based on ERP and behavioral outcomes, which indicated that the differences lay primarily between high and low expectation within attended conditions, time–frequency analysis focused on alpha-band (8–13 Hz) energy differences between high and low expectation under both sham and anodal stimulation. Within the 200–250 ms window, sham stimulation showed significantly lower alpha energy under high expectation compared to low expectation, whereas no significant difference was found under anodal stimulation. These results suggest that 10 Hz tACS applied over the right P4 site reduced the difference between high and low attentional expectation.

This study examined the interaction between temporal attention and expectation under different stimulation conditions. Under sham stimulation, although the two-way interaction between temporal attention and expectation was not statistically significant, post hoc comparisons revealed a classic pattern: a significant behavioral difference between high and low temporal expectation was observed only under attended conditions, but not under unattended conditions. In contrast, under anodal tACS, this attention-dependent differentiation of expectation effects was markedly reduced. To further assess the impact of stimulation conditions, a 2 (stimulation) × 2 (temporal attention) × 2 (temporal expectation) three-way ANOVA was conducted. For reaction time (RT), the three-way interaction was significant, *F*_(1,31)_ = 4.230, *p* = 0.048, *η*^2^ = 0.004, indicating that anodal tACS over the right posterior parietal cortex significantly modulated the interaction between temporal attention and expectation. Simple effect analysis showed that under sham stimulation, the difference between high and low expectation was significant when attention was effective (*F* = 11.880, *p* = 0.002) but not when it was ineffective (*F* = 0.580, *p* = 0.452). Conversely, under anodal tACS, this differential effect was no longer significant (attention effective: *F* = 0.017, *p* = 0.897; attention ineffective: *F* = 1.363, *p* = 0.252). The three-way interactions were not significant for either accuracy or N1 amplitude. In summary, the significant three-way interaction on RT and the subsequent analysis demonstrate that 10 Hz tACS over the right posterior parietal cortex can attenuate the behavioral-level interaction between temporal attention and temporal expectation.

The interaction between temporal attention and temporal expectation appears to primarily influence early perceptual stages rather than later processing stages. The enhanced N1 amplitude observed in the present study is highly consistent with the perceptual gain model of temporal attention, which proposes that the brain transiently increases excitability in sensory cortices at anticipated points in time [32,38,39,40]. One study combined the temporal cueing paradigm with the rapid serial visual presentation (RSVP) technique to maximize the processing demands of perceptual analysis, investigating whether expectation-based attention can similarly modulate perceptual processing. Behavioral results revealed that directing attention to a specific time interval significantly enhanced the efficiency of early perceptual processing [41]. Temporal expectation may contribute to the perceptual gain of temporal attention via a key mechanism: the modulation of alpha oscillation power. The rhythmic inhibitory function of alpha oscillations acts as a gating mechanism. By reducing alpha power (desynchronization) at anticipated moments, the brain enhances the excitability of the visual cortex, thereby applying a ‘gain effect’ to stimuli appearing at expected times and optimizing their perceptual processing [42].

The rPPC is a key hub for integrating sensory and motor information and is crucial for redirecting attention. In tasks requiring attention to timing, people use cues to shift their focus to a specific moment in time [43]. The rPPC is central to converting these sensory cues into plans for action. Research shows that reducing rPPC activity specifically harms the ability to detect stimuli from unexpected locations. This means it affects attention shifting, not basic perception [44]. Therefore, the rPPC plays a key role in quickly and accurately redirecting attention. Although Huang et al. (2014) [45] studied auditory spatial attention and our work focuses on temporal attention, both processes may rely on similar cognitive mechanisms for reorienting attention. Their findings showed that the right posterior parietal cortex (rPPC) uses alpha-band (7–13 Hz) activity to help coordinate sensory information during spatial orienting [45]. This implies that the rPPC and its alpha-band (7–13 Hz) rhythm serve as a supramodal hub in attention control. Accordingly, our observation that alpha-tACS over the rPPC specifically modulates brief temporal attention suggests that this region and its intrinsic rhythm also play a central role in redistributing attention across time.

This study has several limitations. First, it exclusively employed symbolic cues to examine temporal prediction and did not incorporate rhythm-driven temporal expectations; given that cue-based and rhythm-based time expectations rely on distinct neural mechanisms [46], the findings are specific to voluntary, cue-guided temporal expectation and may not generalize to rhythm-driven counterparts. While “temporal expectation” was explicitly defined as the prior probability conveyed via symbolic cues, hazard rate dynamics—the time-dependent change in event likelihood—may have additionally modulated expectation strength in long-interval trials with uncertainty. Specifically, participants implicitly updated the conditional probability of target occurrence as time elapsed when the target did not appear at earlier time points, and this continuous dynamic signal may partly explain why static cue-based expectation effects were harder to capture under long-interval conditions [12,47]. That said, the core manipulation and conclusions of this study remain focused on the discrete, probabilistic temporal predictability established by cue-based paradigms, and future research could adopt more refined designs combined with computational modeling to dissociate the respective contributions of cue-learning and hazard rate inference to temporal expectation formation.

Second, despite using standardized tACS parameters (1.5 mA, 20 min) to ensure reproducibility, these fixed, standardized parameters failed to account for individual anatomical and sensitivity differences, which may cause variability in delivered electric field strength and compromise intervention consistency. Compounded by the physical properties of transcranial electrical stimulation, the electric field inevitably spreads beyond the targeted rPPC and may influence interconnected neural networks; future studies could thus benefit from individualized electric field modeling based on structural MRI to enhance spatial specificity. Finally, we did not administer a condition-guessing questionnaire to participants to assess their ability to distinguish active from sham tACS stimulation, which precludes direct verification of blinding effectiveness and introduces additional interpretive uncertainty to the present findings.

## 5. Conclusions

We provide suggestive evidence that the interaction between temporal attention and expectation may facilitate the allocation of neural resources during early perceptual stages by potentially enhancing cortical excitability specifically during short, rather than long, temporal intervals. This facilitation is reflected in amplified N1 responses and reduced alpha power under high attentional expectation, with the rPPC serving as a key region for integrating temporal predictions with sensory processing. This finding offers valuable insights for studies investigating the neurocognitive mechanisms underlying temporal attention and expectation.

## Data Availability

Due to privacy considerations, the data and materials used and analyzed during the current study are available from the corresponding author on reasonable request.

## Abbreviations

The following abbreviations are used in this manuscript:

tACS	Transcranial Alternating Current Stimulation
tDCS	Transcranial Direct Current Stimulation
EEG	Electroencephalography
ERP	Event-Related Potential
preSMA	Presupplementary Motor Area
PPC	Posterior Parietal Cortex
RT	Reaction Time
ACC	Accuracy
